# One-step solvothermal synthesis of high-emissive amphiphilic carbon dots *via* rigidity derivation†
†Electronic supplementary information (ESI) available: Calculations, photographs, EDS, spectra and cell viability results. See DOI: 10.1039/c7sc04607c


**DOI:** 10.1039/c7sc04607c

**Published:** 2017-12-12

**Authors:** Pei Zhao, Xuping Li, Glib Baryshnikov, Bin Wu, Hans Ågren, Junji Zhang, Liangliang Zhu

**Affiliations:** a State Key Laboratory of Molecular Engineering of Polymers , Department of Macromolecular Science , Fudan University , Shanghai 200433 , China . Email: zhuliangliang@fudan.edu.cn; b Division of Theoretical Chemistry , Biology School of Biotechnology , KTH Royal Institute of Technology , SE-10691 Stockholm , Sweden; c Key Laboratory for Advanced Materials , Institute of Fine Chemicals , East China University of Science and Technology , Shanghai 200237 , China; d Department of Chemistry and Nanomaterials Science , Bogdan Khmelnitsky National University , Cherkasy , 18031 , Ukraine; e Institute of Nanotechnology , Spectroscopy and Quantum Chemistry , Siberian Federal University , 660041 Krasnoyarsk , Russia

## Abstract

In nanoscience, amphiphilic carbon dots (ACDs) are of great importance due to their excellent transferability for application in biological sensing, imaging and labelling. However, facile synthetic strategies are still limited, especially for obtaining high-emissive ACDs. Since the development of a high-emissive feature is strongly desired for improving the practical resolution *in vivo*, here we report a chemical strategy that uses rigid molecules to straightforwardly construct amphiphilic carbon dots (ACDs) with high luminescence quantum yields (QYs). By using 1-[bis(dimethylamino)methylene]-1*H*-1,2,3-triazolo[4,5-*b*]pyridinium 3-oxide hexafluorophosphate (HATU), a typical coplanar compound, as the only precursor, well-defined ACDs were prepared *via* a one-step solvothermal process which exhibited a superior QY of up to 29%, largely superior to those prepared from precursors with less rigid structures. The effect can be mainly attributed to a significant suppression of the competition of non-radiative decay through rigidity derivation. Metal ionic doping during the synthesis resulted in a further improvement of the crystallinity and monodispersity of the materials, with retention of the high-emissive ability. This high-emissive photoluminescence behavior of the ACDs is accompanied with an excitation-wavelength dependence, a high biocompatibility and a low toxicity, which together make the ACDs advantageous for application in multi-channel bioimaging.

## Abstract 2

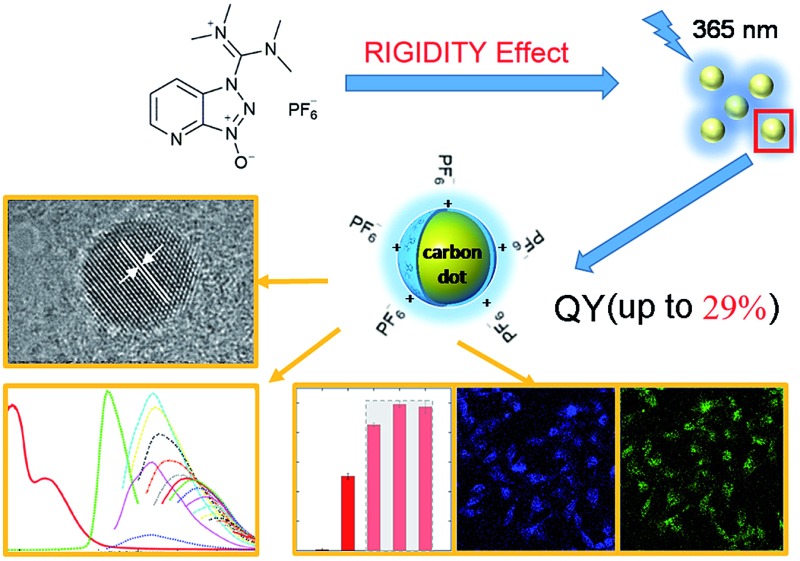
Here we report a chemical strategy that uses rigid molecules to straightforwardly construct amphiphilic carbon dots (ACDs) with high luminescence quantum yields (QYs).

## Introduction

On account of their low toxicity, stable photoluminescence, high biocompatibility and low cost, carbon dots (CDs) have gained increasing research interest since their first serendipitous discovery in 2004.^1^ In particular, amphiphilic carbon dots (ACDs) appear to be superior in the transferability that allows applications in bioimaging,^2^ biosensors,^3^ nanomedicine^4^ and so on.^5^ In the past few years we have witnessed great progress in developing synthetic methodologies for ACDs and the strategies have been largely focused on the employment of polymers, surfactants and ionic liquids as precursors.^6^ However, the luminescence quantum yield (QY) of these acquired ACDs is relatively limited compared to their routine counterparts, probably because the light-emitting process is readily quenched in a charge-separated form of the nanocrystals. Although the radiative decay process has been regulated by a variety of approaches in nanoscience (*e.g.* coating and post-modification, *etc.*) including in the field of CDs,^7^ it remains desirable, albeit a great challenge, to develop easy-to-handle synthetic strategies to directly access high-emissive ACDs.

Since high-emissive systems have been playing a significant role in the promotion of resolution and practicability in applied optoelectronics and *in vivo*, scientists are always seeking different ways to constantly improve material QYs. In the field of organic functional materials, the rigidity of molecular structures is essential to strengthen the radiative decay process by inhibiting competitive energy loss originating from molecular vibration and rotation.^8^ With these facts in mind, we try in this work to apply the concept of molecular rigidity to the synthesis of ACDs in anticipation that ligands or precursors with particular structures can provide effective rigidity derivation towards the formation of nanocrystal cores to facilitate the radiative emission process of the whole system. Moreover, an additional doping strategy to regulate the quality of the prepared nanomaterials is also considered.^9^

**Fig. 1:**
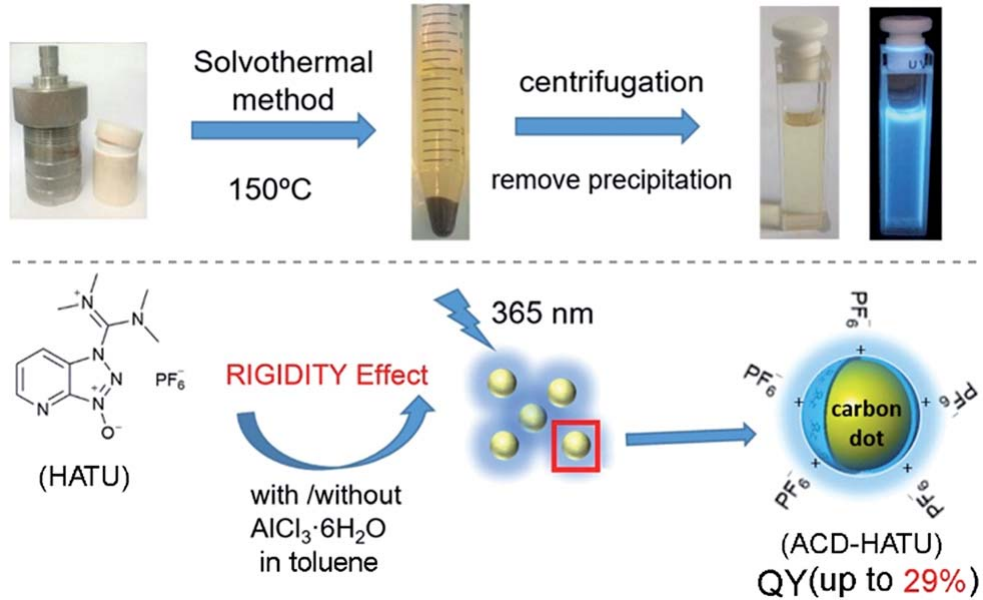
Synthesis of ACDs from the derivation of rigid molecular structures by a one-step solvothermal method with the employment of a coplanar structure HATU as the only precursor. The synthesized ACDs show strong photoluminescence (QY up to 29%) under UV light (excited at 365 nm).

Herein we demonstrate the one-step solvothermal synthesis of ACDs from rigid molecular structures as the only precursors (see the illustration in Fig. 1). Generally, CDs can be synthesized by different “top-down”^1^,^10^ and “bottom-up” approaches.^11^ Solvothermal treatment is a cheap, environmentally friendly and nontoxic technique to produce CDs.11*e* In our work, a series of commercially-available heteroatomic compounds were used under relatively mild reaction conditions to conduct a rapid and facile synthesis to produce ACDs, and the results were analyzed from the perspective of rigidity derivation (see the detailed procedures in the Experimental section). We show that 1-[bis(dimethylamino)methylene]-1*H*-1,2,3-triazolo[4,5-*b*]pyridinium 3-oxide hexafluorophosphate (HATU) can serve as an ideal rigid precursor to form ACDs with high QYs, and that the crystallinity and monodispersity can be further improved by metal ionic doping. While the leading ACDs can also reveal an excitation-wavelength dependent emission, our materials can achieve direct application in multi-channel cellular imaging, due to their additional superior biocompatibility and low biotoxicity. Such a preparation strategy might lead to a new concept for advancing fundamental nanoengineering to address high-tech light-emitting materials of different size scales.

## Experimental

### Chemicals and reagents

1-[Bis(dimethylamino)methylene]-1*H*-1,2,3-triazolo[4,5-*b*]pyridinium 3-oxide hexafluorophosphate (C_10_H_15_OF_6_N_6_P, HATU, 99.5%), AlCl_3_·6H_2_O (98%), diisopropylamine (98%), benzyltriethylammonium chloride (99%), 1-benzylimidazole (98%), toluene (99.5%), ethanol (99.7%), acetonitrile (99%), *N*,*N*-dimethylformamide (DMF, 99.5%) and pyridine (99.5%) were purchased from Energy Chemical, Vetec™, Alfa Aesar, Shanghai Dahe Chemical and Tansoole Company. All reagents were of analytical or reagent grades and used without further purification. Deionized water (18.2 MΩ cm) was obtained from a F’DEER water purification system.

### Synthesis

HATU (0.5 mmol) or other precursors (0.5 mmol benzyltriethylammonium chloride, 0.5 mmol 1-benzylimidazole, 1 mL diisopropylamine) were added with stirring at room temperature to poly(tetrafluoroethylene) (Teflon) lined flasks (50 mL) containing 30 mL solvent. After 20 minutes of stirring they were transferred into a stainless steel autoclave and put in the oven. The autoclaves were heated up to 150 °C, maintained at this temperature for 8 hours, and cooled to room temperature naturally in the oven. The yellowish solution and dark brown oily precipitate were separated by centrifugation. The doped materials were prepared in the same way only with AlCl_3_·6H_2_O (1 mmol) added.

#### TEM

The (high-resolution) transmission electron microscopy, (HR)TEM, images were obtained on a JEOL JEM-2100F field emission electron microscope with 200 kV field emission. The mother liquids were dissolved in 1 mL alcohol. The solutions were then dropped on an ultra-thin carbon membrane coated copper grid and the excess of the solution was removed with filter paper.

#### AFM

Atomic force microscopy (AFM) was conducted in tapping mode on a Bruker Multimode-8.

#### EDS

Energy dispersive X-ray spectroscopy (EDS) was carried out on an Ultra 55 field emission scanning electron microscope equipped with an EDS detector from Zeiss Company.

#### Raman

Raman spectroscopy was carried out using ACDs in aqueous droplets on the slide. The spectra were recorded on a Horiba Jobin Yvon XploRA spectrometer equipped with a 10× objective and a laser with a wavelength of 532 nm. Data were collected between 850 cm^–1^ and 1700 cm^–1^ using a grating of 600 grooves per mm. They were normalized using the LabSpec Version 5 software package.

#### FTIR

Fourier transform infrared (FTIR) spectroscopy was carried out with a Thermofisher Nicolet 6700 spectrometer using KBr pellets as the sample matrix in the wavenumber range of 400–4000 cm^–1^.

#### XPS

X-ray photoelectron spectroscopy (XPS) was performed on a PHI5300 using a magnesium Kα source (250 W, 14 kV).

#### UV-vis

The ultraviolet-visible (UV-vis) absorption spectra were measured in the region of 250–800 nm on a Shimadzu 1800 spectrophotometer.

#### PL

The photoluminescence (PL) excitation and emission spectra, and the lifetime and quantum yield, were recorded on a Shimadzu RF-5301, FLS 920 (Edinburgh Instruments) and a QM40 spectrofluorophotometer from Photo Technology International (PTI), respectively.

The quantum yields were determined using an integrating sphere. In this approach the absolute photoluminescence quantum yield is given by*Φ* = [*E*_in_(*λ*) – (1 – *α*)*E*_out_(*λ*)]/[*X*_empty_(*λ*)*α*]*α* = [*X*_out_(*λ*) – *X*_in_(*λ*)]/*X*_out_(*λ*)

In these equations, *E*_in_(*λ*) and *E*_out_(*λ*) are the integrated luminescence as a result of direct excitation of the sample and secondary excitation, respectively. The latter emission is due to reflected excitation light from the sphere walls hitting the sample. *X*_empty_(*λ*) is the integrated excitation profile with the empty sphere. *α* is the film absorptance. *X*_in_(*λ*) is the integrated excitation when the sample lies directly in the excitation path and *X*_out_(*λ*) is the integrated excitation when the excitation light first hits the sphere wall as previously explained. All of the spectra were recorded with the same excitation and emission monochromator bandpass.^12^

The relative quantum yields were determined in raw solvent using optically matching ethanol solutions of rhodamine B (*Φ*_r_ = 0.65) and quinine sulfate in 0.1 M H_2_SO_4_ (*Φ*_r_ = 0.54) as references. The quantum yields were calculated using the below equation:*Φ*_f_ = *Φ*_r_(*A*_r_*F*_s_/*A*_s_*F*_r_)(*η*_s_^2^/*η*_r_^2^)where *A*_s_ and *A*_r_ are the absorbance of the sample and reference solutions, respectively, at the same excitation wavelength, *F*_s_ and *F*_r_ are the corresponding relative integrated emission intensities, and *η* is the refractive index of the solvent.^13^

### Cell labeling and cytotoxicity assay

The cell viability was quantitatively determined by the Cell Counting Kit-8 (CCK-8) assays. Hela cells were obtained from the Cell Bank of the Chinese Academy of Science (Shanghai, China) and seeded onto a 96-well cell culture plate at a density of 1 × 10^4^ cells per well in Dulbecco’s modified Eagle medium (DMEM) containing 10% FBS under 5% CO_2_ at 37 °C. After the cells grew for 12 h, the medium was changed into a new medium (200 μL per well) containing the carbon dots at 1000, 500, 100 and 50 μg mL^–1^. After the cells were incubated with the sample for 24 h, the medium was replaced with 100 μL of fresh medium. Subsequently, 10 μL of CCK-8 was added to each well and homogeneously mixed, followed by incubation at 37 °C for 4 h in a CO_2_ incubator, and finally, 80 μL of the solutions were put into a new 96-well plate. The optical density of each well at 450 nm was read using a microplate reader.

Hela cells were seeded in 35 mm plastic-bottomed dishes and grown in DMEM medium for 24 h, then the cells were incubated in fresh medium containing 100 μg mL^–1^ ACDs for another 4 h. The cells were washed with a phosphate buffer saline (PBS) solution (pH 7.4) three times and fixed with paraformaldehyde at 4 °C for 15 min. The luminescence images of the cells were captured using a Nikon laser scanning confocal microscope C2^+^ with different channels.

### Computational details

The structures of the precursor compounds and their isomers have been optimized at the density functional theory (DFT) level using the B3LYP^14^ hybrid exchange-correlation functional and the extended 6-311++G(d,p) basis set.^15^ The same method has been used to determine the true minimum of the total energy and corresponding vibrational frequencies. For the optimization procedure we have used the “tight” criterion for self-consistence field (SCF) convergence starting from the different initial conformations of these compounds. All the detected isomers (conformers) with a true minimum on the potential energy surface (PES) are presented in Fig. 1 and S1.† Among them HATU and BTEAC were considered in the cationic form while counterions were omitted for clarity. The basic principle applied for the conformational analysis was a gradual rotation of the main functional groups along the corresponding σ-bonds with the subsequent optimization procedure. All DFT calculations have been performed using the GAUSSIAN16 (ref. 16) software. The QTAIM calculations^17^ have been realized within the AIMAll program package.^18^

## Results and discussion

In our work, ACDs were synthesized by solvothermal reactions from a series of precursors dissolved in toluene at 150 °C for 8 h. The heteroatomic precursors include HATU, diisopropylamine (DIPA), benzyltriethylammonium chloride (BTEAC) and 1-benzylimidazole (BI), which were chosen due to their close molecular length and polarity to investigate the effect of molecular coplanarity and rigidity. All these precursors can produce ACDs with similar compositions and sizes under the same reaction conditions. However, by using these molecules for derivation, we observed that HATU leads to the highest luminescence QY (up to 29% in toluene) in the prepared ACDs (ACD–HATU) whereas the other precursors only yield ACDs (ACD–DIPA, ACD–BTEAC and ACD–BI) with QYs below 2% (0.6–1.7%).

**Fig. 2:**
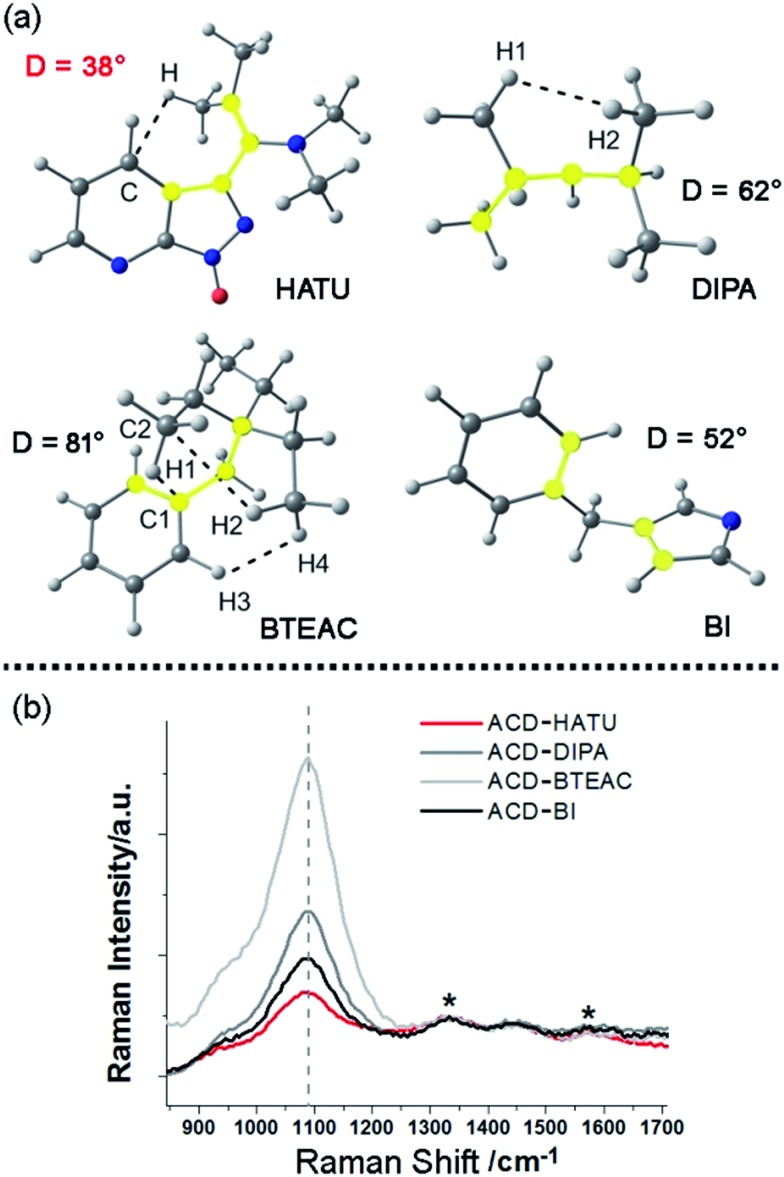
Study of the molecular rigidity of the precursors: (a) coplanarity parameters in the optimized geometries obtained by B3LYP/6-311++G(d,p) of the precursor molecules. The dashed lines indicate the non-covalent interactions detected by AIM analysis of the electronic density distribution function. The atoms that compose the highlighted dihedral angles are shown in yellow. (b) Normalized Raman spectra of the ACDs in water. The internal standard signals are marked with “*”.

To gain the precise structural information of these precursors for comparison, computational analysis was firstly employed upon their optimized geometries. Since a coplanarity parameter can straightforwardly reflect the molecular rigidity, we measure the dihedral angle in these optimized geometries between the polar C–N bond and the main bulk group, as shown in Fig. 2a. Due to the formation of an intermolecular hydrogen bond, the dihedral angle in HATU (38°) turned out to be smallest among these precursor structures. On the other hand, HATU bears more sp^2^ hybrid orbitals in its main aromatic skeleton than the other molecules. These results suggest that normally HATU will reveal the biggest conformational rigidity in comparison with the rest of this series of precursors. In contrast, the lack of coplanarity and the existence of different conformers (see Fig. S1†) readily make the other precursor molecules less rigid.

Furthermore, direct experimental evidence to confirm the rigidity derivation effect on the prepared ACDs can also be obtained from the Raman spectra. CDs generally exhibit a D-band (∼1330 cm^–1^) and a G-band (∼1600 cm^–1^).^19^ In our case, a strong Raman peak, assigned to C–N stretching vibrations (*V*_C–N_, ∼1080 cm^–1^), was also observed.^20^ Since it would be understandable that the carbon core has a minimum sensitivity to the amphiphilic molecular structures at the rim of the nanosystems, the D-band and G-band were set as the internal standard to compare the normalized Raman spectra among these ACDs (Fig. 2b). Although these synthesized ACDs generally exhibit a similar size and number of atomic bonds, we find that the intensity of *V*_C–N_ shows a great difference relative to the internal standard. The *V*_C–N_ in ACD–HATU is the weakest one, indicating that the peripheral vibration behavior was significantly restricted. In terms of the above geometrical studies, we can conclude that the high coplanarity and strong rigidity effect of HATU greatly reduced molecular vibrations, so as to inhibit competitive energy loss and non-radiative decay and allow a remarkable enhancement of the QY of the whole nanocrystal. Since HATU itself cannot emit any luminescence (see the photographic comparison in Fig. S2†), the as-prepared ACD with high QY can attract unique interest as a single nanoplatform. With these ideas in mind, we turn to explore the structural and photophysical features of ACD–HATU in detail.

**Fig. 3:**
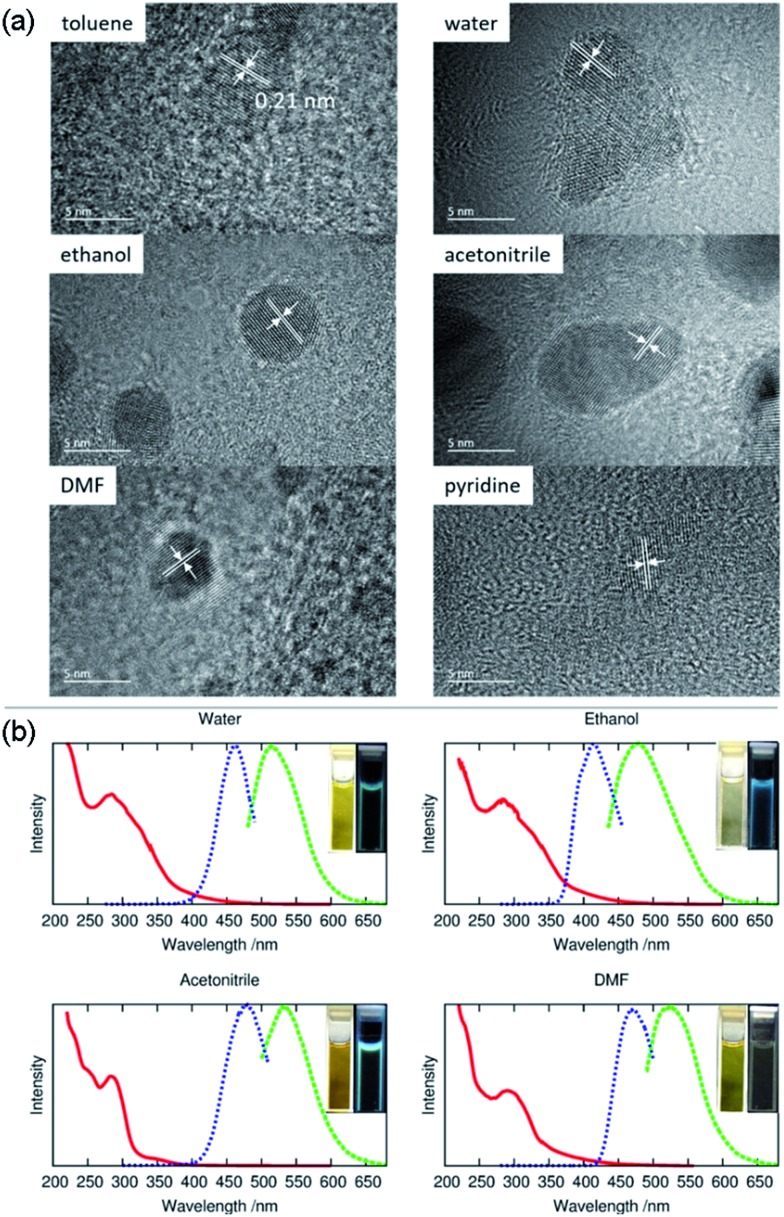
Morphological characterization and optical studies: (a) high resolution transmission electron microscopy (HRTEM) images, (b) the normalized optical absorption spectra (red curve), photoluminescence excitation (blue curve) and photoluminescence emission (green curve) spectra of the carbon dots prepared by solvothermal methods with HATU in toluene, water, ethanol, acetonitrile, *N*,*N*-dimethylformamide (DMF) and pyridine, respectively.

Meanwhile, the FTIR spectrum was recorded to obtain more structural information of ACD–HATU, as shown in Fig. S3.† To further compare and investigate the role of HATU, we also replaced toluene with other solvents to monitor the environmental effect. The TEM and AFM images with associated height analysis (Fig. S4 and S5†) show that these dots have a spherical nanoshape with diameters in the range of 2–5 nm. Fig. 3a shows HRTEM images of ACD–HATU obtained from different solvents. The HRTEM suggests that these CDs are crystalline, which can be proven by the oriented lattice fringes in an enlarged view. The fringe separations are calculated to be ∼0.21 nm which are close to the (100) diffraction plane of graphite.

The energy dispersive X-ray spectroscopy (EDS) results (Fig. S6 and Table S2†) for ACD–HATU obtained from toluene show the peaks of the elements C, O, N, P and F. The ratio of P to F (1 : 6) indicates the existence of PF_6_^–^, further suggesting an amphiphilic species from the structural aspect. The X-ray photoelectron spectroscopy (XPS) survey reveals that the sample consists mainly of carbon, nitrogen, oxygen and fluorine with a C 1s peak at *ca.* 287 eV, a N 1s peak at *ca.* 401 eV, an O 1s peak at *ca.* 533 eV and a F 1s peak at *ca.* 691 eV. In detail, the C 1s spectrum shows two peaks at 285.7 eV and 288.2 eV which are attributed to C–C and C–O, respectively. The O 1s spectrum shows two peaks at 532.2 eV and 534.0 eV, corresponding to C–OH and C–O–C, respectively (Fig. S7†).

The UV-vis spectra in Fig. 3b show absorption peaks around 290 nm and 350 nm. The peaks around 290 nm indicate the presence of the C=C bonds and the C=O bonds, which further signify the formation of a CD core. The transition at 350 nm reflects the existence of the surface connected chemical groups. The corresponding PL spectra were recorded according to their excitation to display emissions around 470 nm, 510 nm, 475 nm, 530 nm and 520 nm while in toluene, water, ethanol, acetonitrile and DMF, respectively. The emission of carbon dots normally undergoes a red shift upon an increase of solvent polarity according to previous reports.^21^ However, the PL in our case does not follow this trend. From the HRTEM images, we can see that the morphology of the samples obtained from DMF, water and acetonitrile were in an aggregated form with two or three original dots gathered together. The size of the single dots obtained from varied solvent conditions remains identical, only the solvent attachment on the surface alters the conjugation degree of the sp^2^ carbons^22^ so as to result in different emission, although the fluorescence shifts are dominated mainly by the quantum confinement effect.

Even using water, we found that the solution still revealed an emission resolvable by the naked eye under the 365 nm excitation UV lamp. The fluorescence can be also proven from photoluminescence measurements (Fig. 3b). The relatively reduced emission can be attributed to an aggregation-caused quenching (ACQ) effect.^23^ In water, acetonitrile and pyridine, a few of the dots are aggregated together while in toluene, ethanol and DMF the dots are well-dispersed. Their relative quantum yields (quinine sulfate in 0.1 M H_2_SO_4_ as the reference) of the ACDs were ∼7%, ∼2%, ∼20% and ∼2% prepared from water, ethanol, acetonitrile and DMF, respectively. Anyway, the high-emissive characteristic of the ACDs can still ensure effective bio-imaging as demonstrated below. To give direct evidence for the amphiphilic property of the carbon dots, we also mixed the carbon dots prepared in toluene with deionized water, ultrasonicated them for two minutes and kept them for a couple of hours until the solution separated into two clear phases. Under the 365 nm UV lamp, both the lower aqueous and upper organic phases exhibited blue luminescence (Fig. S2c and S2d†) which means that a proportion of the carbon dots were transferred from the toluene to water sue to their amphiphilicity.

**Fig. 4:**
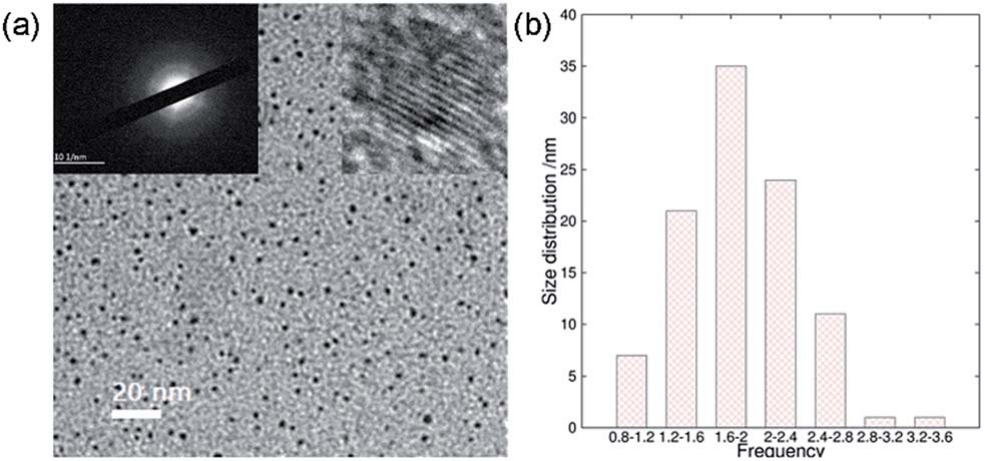
Improved crystallinity and monodispersity due to metal ionic doping: (a) transmission electron microscopy (TEM) image with inset images of the selected area electron diffraction (SAED, left) and the high-resolution TEM image (right), and (b) the size distribution calculation based on one hundred dots of ACD–HATU doped with Al.

In addition to the QY, metal ionic doping also brought about improved crystallinity and monodispersity during the formation of the ACDs. A series of metal ions were tried for the doping during the synthesis of ACD–HATU, and the emissive property can generally be retained. The mother liquid of ACD–HATU after aluminum doping was centrifuged, and the deposit in the bottom was dropped onto a carbon membrane coated copper grid. This grid can be used on TEM-EDS for elemental determination. The mapping figures (Fig. S8†) showed the existence of aluminum. The doped sample exhibited a spherical shape, with diameters of 0.8–3.6 nm and well dispersed dots with a narrow size distribution as well as more concentrated dots in the same area, as compared with those from the non-doped materials (Fig. 4a and b). More interestingly, the doped ACDs show a larger Stokes shift (∼100 nm) than the undoped ones (Fig. S9†). Doping definitely minimized the self-quenching effect, leading to a longer PL lifetime of 6.7 ns (as compared with the average lifetime of 2 ns for the undoped ACDs, see Fig. S10 in the ESI†), while maintaining a QY that is still high (*ca.* 20%).

**Fig. 5:**
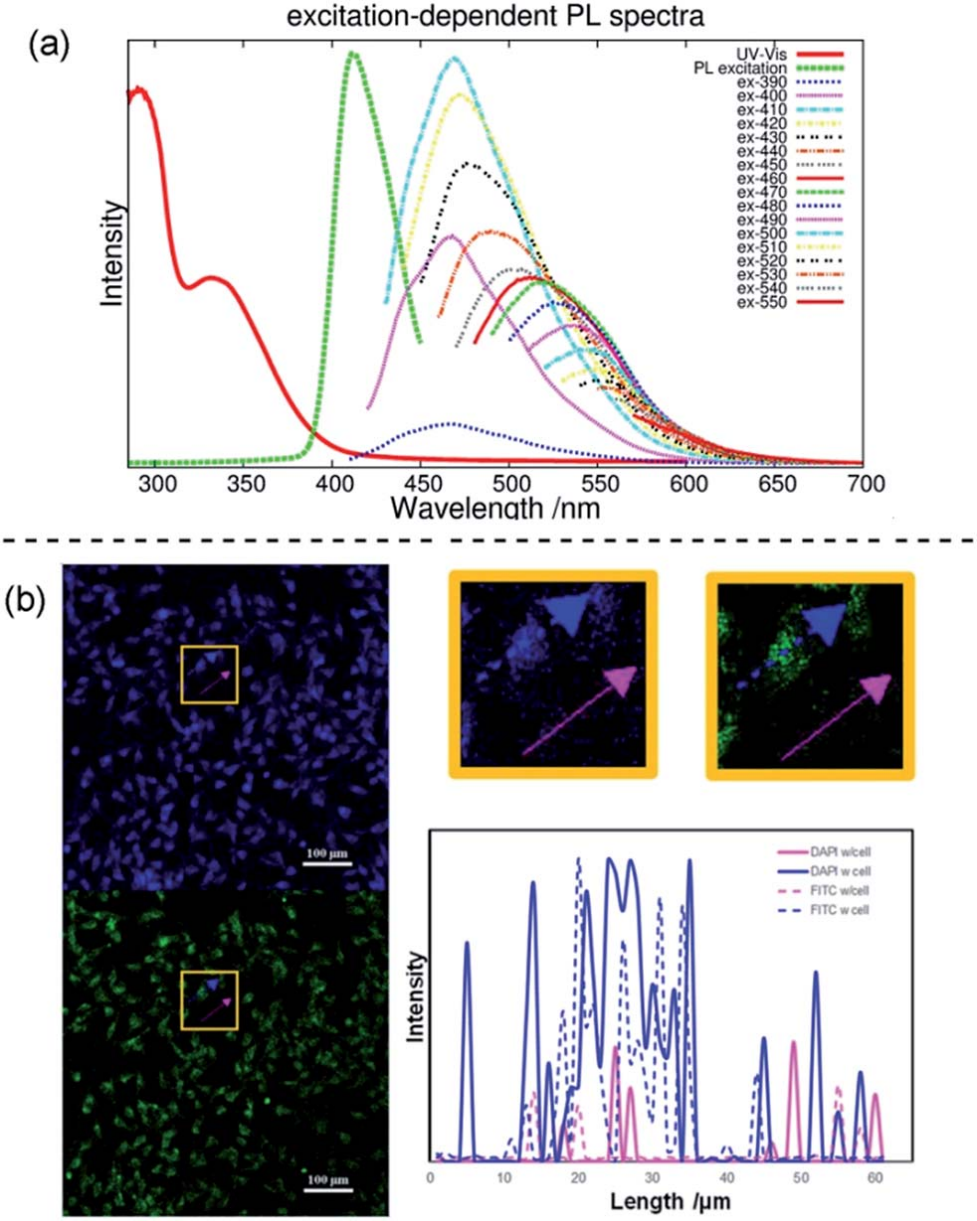
Multi-channel cellular imaging studies. (a) UV-vis, photoluminescence excitation and emission spectra of ACD–HATU with increasing *λ*_ex_ from 390 nm in 10 nm increments. (b) Selective dual-channel imaging: representative results of the confocal fluorescence microphotographs of Hela cells incubated with ACD–HATU, *λ*_ex_ = 408 nm (blue, DAPI Channel) and *λ*_ex_ = 488 nm (green, FITC Channel), and the brightness intensity readout curves along the arrows (the blue color and pink color show the paths going through a cell or not, respectively).

A narrow emission peak is usually one of the excellent PL properties in nanocrystals,^24^ however, CDs can display a unique excitation-dependent PL behavior.^25^ To further explore the fluorescence properties of our ACDs from this perspective, we carried out a detailed study varying the excitation wavelengths from 300 to 550 nm (see Fig. 5a and S11†). Upon excitation from 300 nm to 410 nm, the maximum emission peak wavelength of the ACDs remained unchanged, only accompanied by a progressive increase in the emission intensity. Such a trend is in agreement with the maximum photoluminescence excitation at 410 nm. Furthermore, as expected, the emission peak position underwent a bathochromic shift with a gradual decrease of intensity when the excitation wavelength was increased from 410 nm to 550 nm. These results suggest that an excitation-wavelength dependent PL characteristic can also be shown in our nanomaterials, allowing an exceptional multi-channel imaging effect as exemplified in the cellular studies below.

Although fluorescent probe techniques have been applied in bioimaging for years, demonstrations of the imaging of a single system under the control of detection channels are still scarce.^26^ The CDs we obtained are amphiphilic so that they are soluble directly in water and can be used as a biolabeling reagent. The mother liquid was freeze-dried, mixed with deionized water *via* ultrasonic treatment and centrifuged to separate the precipitate and water. The aqueous system containing ACD–HATU was then used for the cellular experiments. Fig. S12a† shows the viability of the Hela cells using four parallel CCK-8 experiments. At concentrations up to 500 μg mL^–1^, the cell viability exceeds 85%, whereas it exceeds 95% with a concentration of 100 μg mL^–1^. Such a progression suggests that the ACDs can be well endocytosed with low cytotoxicity. The cell spreading morphology is quite normal after incubation (see the bright field image in Fig. S12†), further indicating a favorable biocompatibility of the ACDs since this concentration and exposure time of the ACDs did not cause cell damage and apoptosis.

The effect of multi-channel imaging can be detected by confocal microscopes. As shown in Fig. 5b and S12,† the cell imaging using the ACDs can be clearly observed from both a DAPI channel under 408 nm excitation and an FITC channel under 488 nm excitation. The photoluminescence intensity along the cellular profile can also be quantified (see Fig. 5b), showing that the imaging works well from the distinct signal readouts. Meanwhile, the signal difference with and without going through a cell unambiguously signified the high-resolution of the imaging when using these nanomaterials with high QYs. As we demonstrated above, the ACDs can undergo a remarkable excitation-dependent PL behavior. The investigation suggests that this performance can also occur at the cellular level, in agreement with the corresponding phenomena in solution. Such a spectrally tunable material might thus present a promising selective potential for advancing sensing or labeling at the nanoscale.

## Conclusions

In summary, we have demonstrated a successful one-step solvothermal synthesis of high-emissive amphiphilic carbon dots from HATU, a typical coplanar compound, as the only precursor. A high luminescence quantum yield of the ACDs was observed, as well as a further improvement of crystallinity and monodispersity by metal ionic doping, in which the derivation of the rigid precursor structure, causing suppression of competitive energy loss, played a key role. Our materials, which are biocompatible with a low cytotoxicity, can also be applied *in vivo* to achieve high-resolution multi-channel cellular imaging, relying on an excitation-wavelength dependent emission behavior. We believe that the synthetic strategy demonstrated herein can be valuable for the development of next-generation light-emitting materials at the nanoscale for versatile applications with high brightness.

## Conflicts of interest

There are no conflicts to declare.

## Supplementary Material

Supplementary information
