# Supplementary material for: One-step solvothermal synthesis of high-emissive amphiphilic carbon dots via rigidity derivation
Source: Chem Sci. 2017 Dec 12;9(5):1323–9. doi: 10.1039/c7sc04607c (PMC5887100; doi:10.1039/c7sc04607c)
Supplement: Supplementary file 1 [file SC-009-C7SC04607C-s001.pdf]

Electronic Supplementary Material (ESI) for Chemical Science.
This journal is © The Royal Society of Chemistry 2017

Electronic Supplementary Information

One-Step Solvothermal Synthesis of High-Emissive Amphiphilic Carbon Dots via Rigidity Derivation

Pei Zhao,[a] Xuping Li,[a] Glib Baryshnikov,[b,d] Bin Wu,[a] Hans Ågren,[b,e] Junji Zhang[c] and Liangliang Zhu[a,*]

[a] *State Key Laboratory of Molecular Engineering of Polymers, Department of Macromolecular Science, Fudan University, Shanghai 200433, China. E-mail: zhuliangliang@fudan.edu.cn*
[b] *Division of Theoretical Chemistry and Biology School of Biotechnology, KTH Royal Institute of Technology, SE-10691 Stockholm, Sweden*
[c] *Key Laboratory for Advanced Materials & Institute of Fine Chemicals, East China University of Science and Technology, Shanghai 200237, China*
[d] *Department of Chemistry and Nanomaterials Science, Bogdan Khmelnitsky National University, Cherkasy, 18031, Ukraine*
[e] *Institute of Nanotechnology, Spectroscopy and Quantum Chemistry, Siberian Federal University, 660041 Krasnoyarsk, Russia*

Table of contents in Supporting Information:

Figure S1. Optimized structure for the global and local energy minima of compounds DIPA and BTEAC obtained by B3LYP/6-311++G (d, p) calculations in a gas phase approximation (relative total energies are presented in parentheses in kcal/mole).

Figure S2. Photograph under a 365-nm UV lamp of (a) a HATU solid, (b) HATU solution, (c) ACD-HATU solution in toluene and (d) ACD-HATU solution in water.

Table S1. Calculated frequencies of first normal vibrational mode of the precursor compounds.

Figure S3. Fourier transformed infrared (FTIR) spectroscopy of ACD-HATU.

Figure S4. Transmission electron microscopy (TEM) image (left) and the size distribution calculation based on fifty dots of ACD-HATU in ethanol as an example.

Figure S5. Atomic force microscope (AFM) image and the height profile along the line (inset) of ACD-HATU.

Figure S6. Energy Dispersive X-ray spectroscopy (EDS) image and elemental figure of ACDs.

Table S2. Energy Dispersive X-ray spectroscopy (EDS) elemental analysis results.

Figure S7. The X-ray photoelectron spectroscopy (XPS) and C 1s, O 1s, N 1s, F 1s spectra of of ACD-HATU.

Figure S8. Transmission electron microscopy (TEM)-energy dispersive X-ray spectroscopy (EDS) image (left) of Al doped ACD-HATU and mapping for aluminum (right).

Figure S9. The normalized optical absorption spectra (red curve), photoluminescent excitation (blue curve) and photoluminescent emission (green curve) spectra of Al doped ACDs.

Figure S10. The time-resolved decay spectra and the fitting curves which calculate fluorescence lifetimes of carbon dots obtained in different solvents.

Figure S11. UV-Vis, photoluminescent excitation and emission spectra of carbon dots with increasing λ_{ex} from 300 nm with 10 nm increments.

Figure S12. a) Cell viability of Hela cells incubated with different concentration carbon dots via CCK-8 assay and confocal fluorescence microphotographs of Hela cells incubated with ACD@HATU: b) λ_{ex} = 408 nm, c) λ_{ex} = 488 nm and d) bright field.

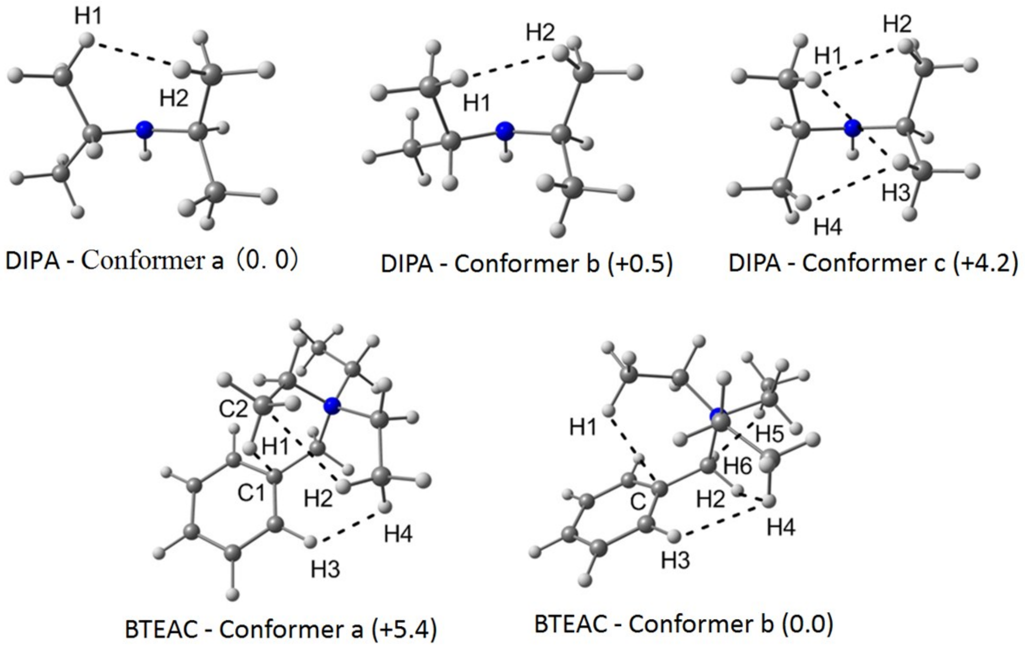

Figure S1. Optimized structure for the global and local energy minima of compounds DIPA and BTEAC obtained by B3LYP/6-311++G (d, p) calculations in a gas phase approximation (relative total energies are presented in parentheses in kcal/mole). Dashed lines indicate the non-covalence interactions detected by AIM analysis of electronic density distribution function.

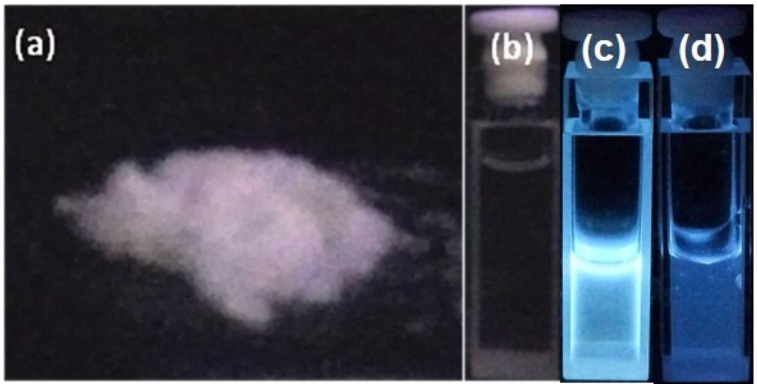

Figure S2. Photograph under a 365-nm UV lamp of (a) a HATU solid sample, (b) HATU solution, (c) ACD-HATU solution in toluene and (d) ACD-HATU solution in water.

Table S1. Calculated frequencies of first normal vibrational mode of the precursor compounds.

	ω_1(HATU), cm^{-1}	ω_1 (DIPA) , cm^{-1}	ω_1 (BTEAC) , cm^{-1}	ω_1 (BI) , cm^{-1}
Conformer a	53.51	45.57	37.3	21.68
Conformer b		45.65	40.87	
Conformer c		57.82		

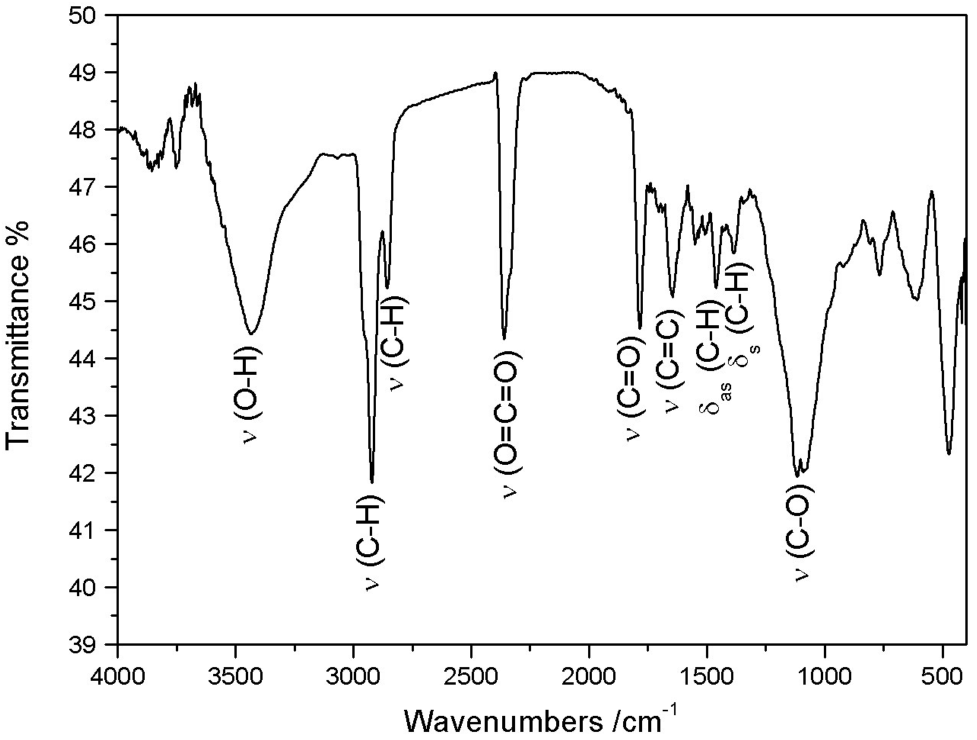

Figure S3. Fourier transformed infrared (FTIR) spectroscopy of ACD-HATU.

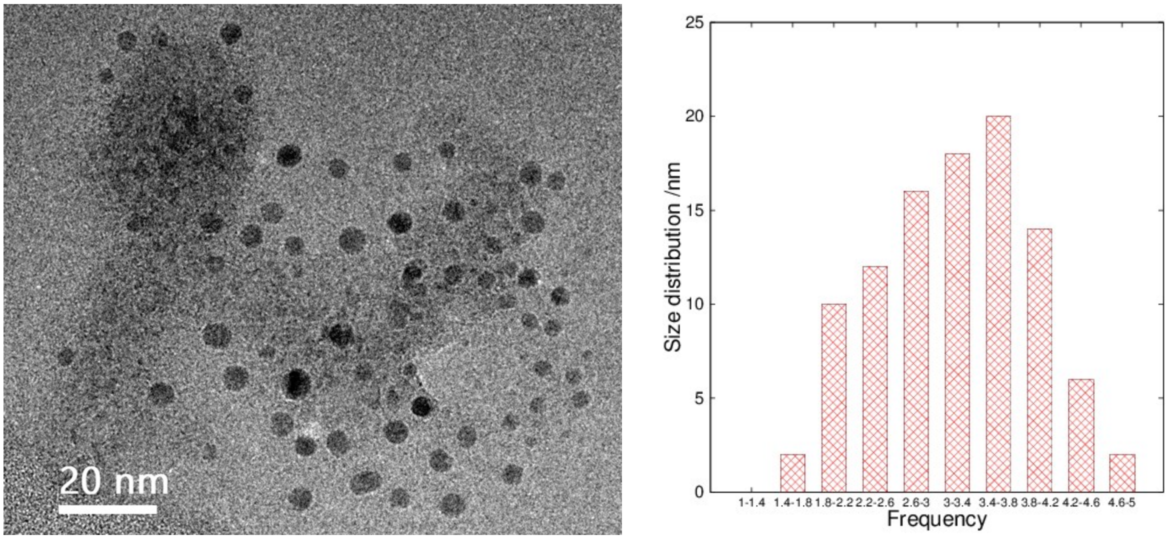

Figure S4. Transmission electron microscopy (TEM) image (left) and the size distribution calculation (right) based on fifty dots of ACD-HATU in ethanol as an example.

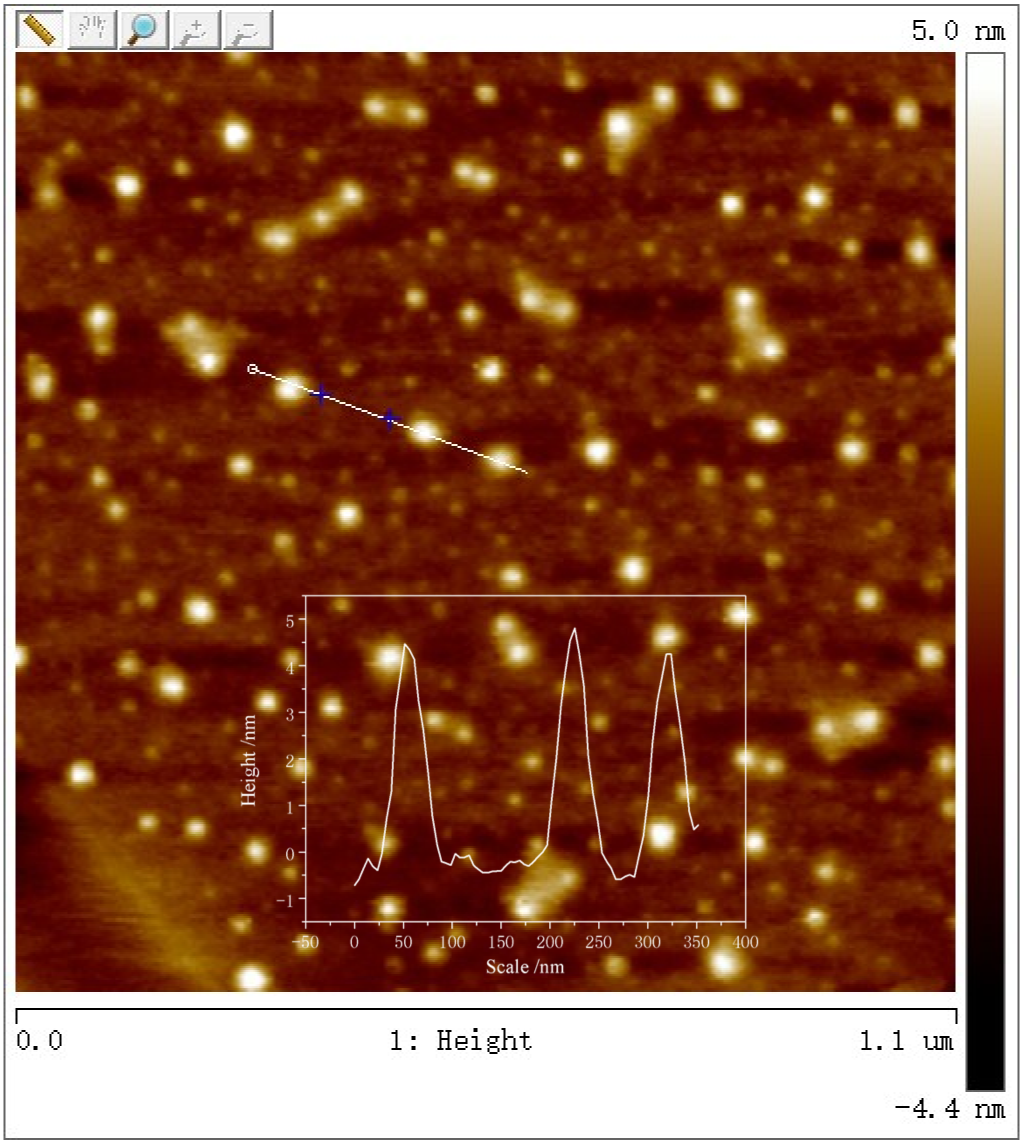

Figure S5. Atomic force microscope (AFM) image and the height profile along the line (inset) of ACD-HATU.

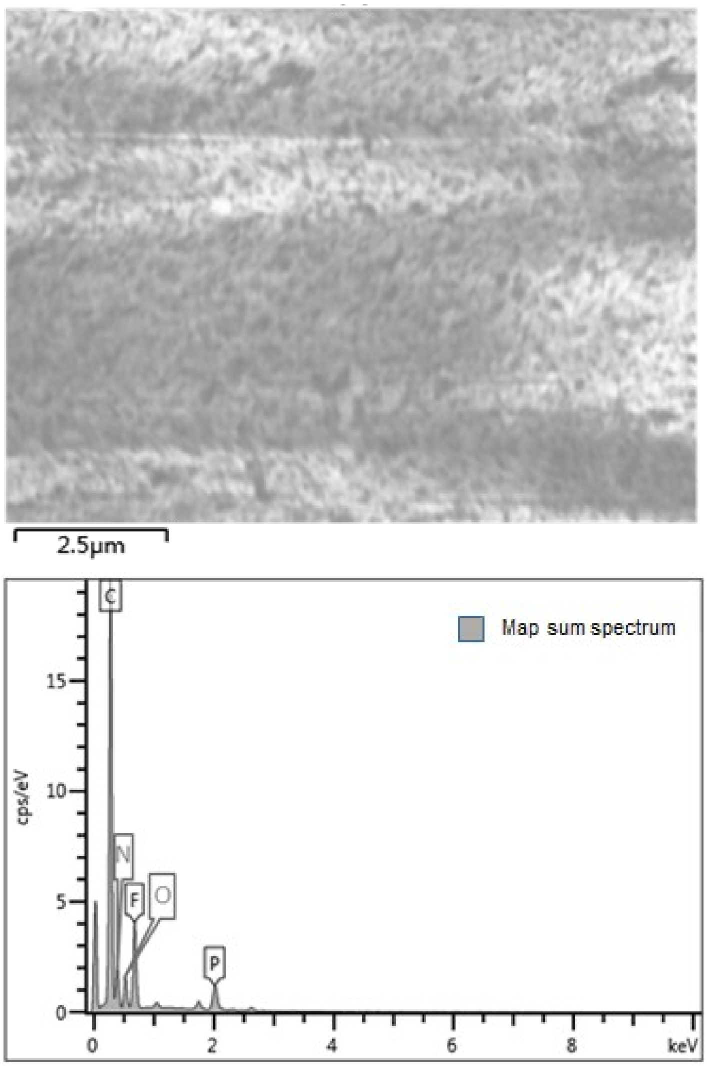

Figure S6. Energy Dispersive X-ray spectroscopy (EDS) image and elemental figure of ACDs.

Table S2. Energy Dispersive X-ray spectroscopy (EDS) elemental analysis results.

Elements	Line type	k ratio	weight%	weight% error	Atom%	standards
C	K line	0.45553	76.64	0.24	84.98	C Vit
F	K line	0.08072	18.37	0.22	12.88	CaF2
P	K line	0.03766	5.00	0.12	2.15	GaP
Total:			100.00		100.00	

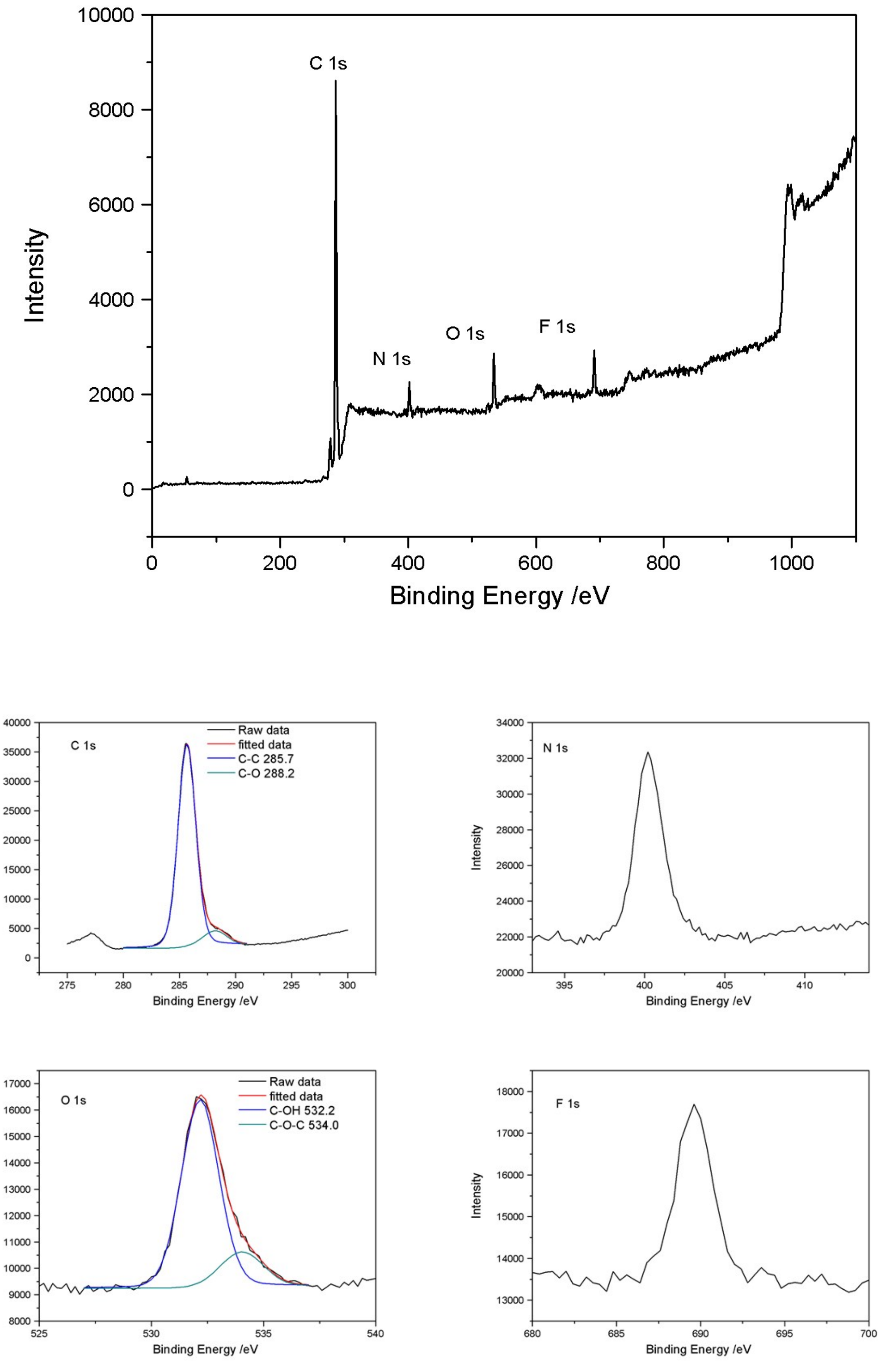

Figure S7. The X-ray photoelectron spectroscopy (XPS) and C 1s, O 1s, N 1s, F 1s spectra of ACD-HATU.

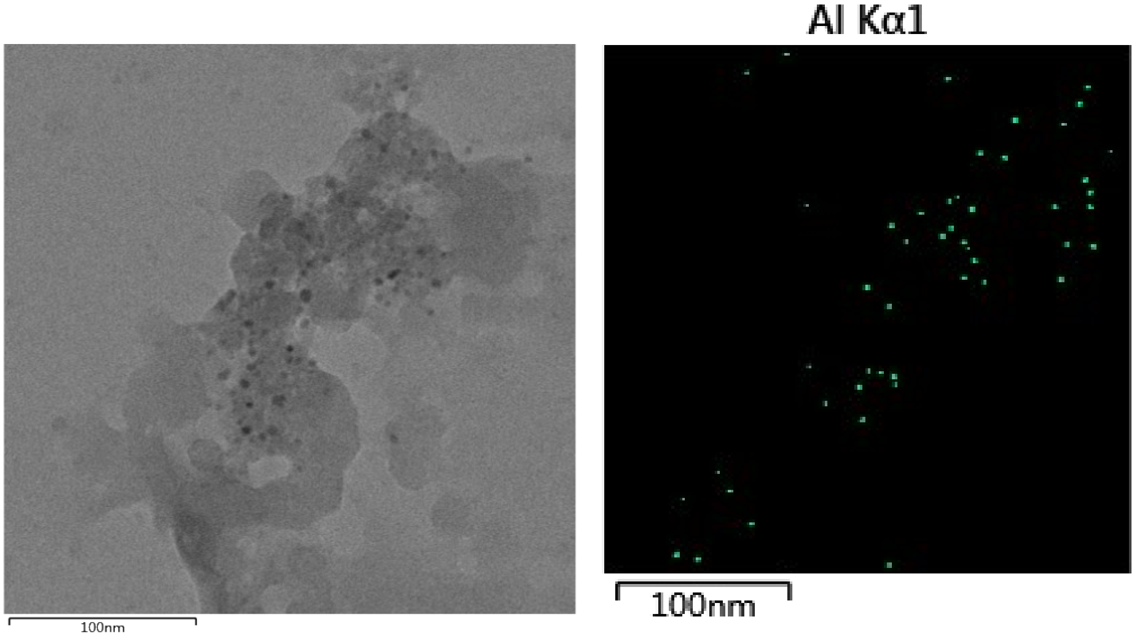

Figure S8. Transmission electron microscopy (TEM)-energy dispersive X-ray spectroscopy (EDS) image (left) of Al doped ACD-HATU and the mapping for aluminum (right).

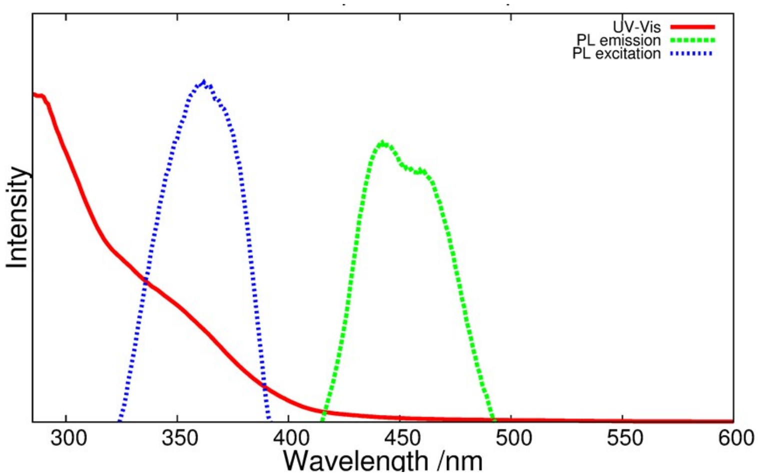

Figure S9. The normalized optical absorption spectra (red curve), photoluminescent excitation (blue curve) and photoluminescent emission (green curve) spectra of Al doped ACDs.

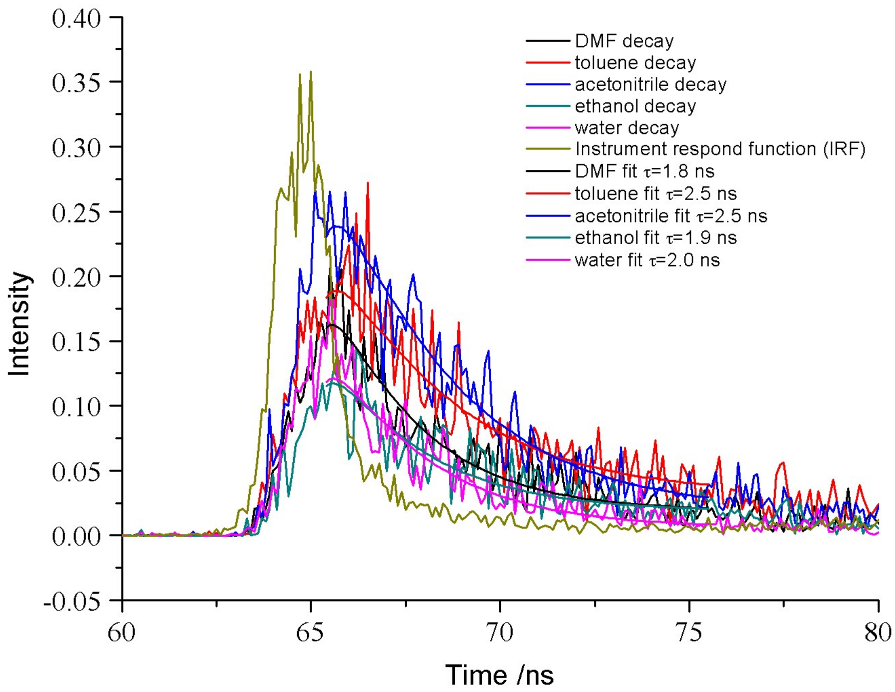

Figure S10. The time-resolved decay spectra and the fitting curves which calculate fluorescence lifetimes of carbon dots obtained in different solvents.

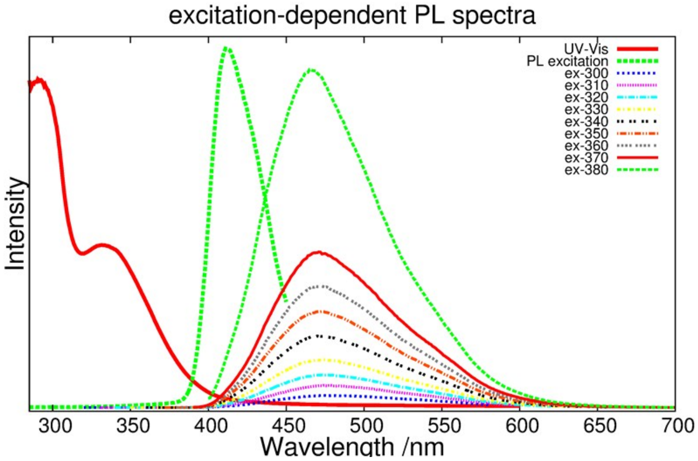

Figure S11. UV-Vis, photoluminescent excitation and emission spectra of carbon dots with increasing λ_{ex} from 300 nm with 10 nm increments.

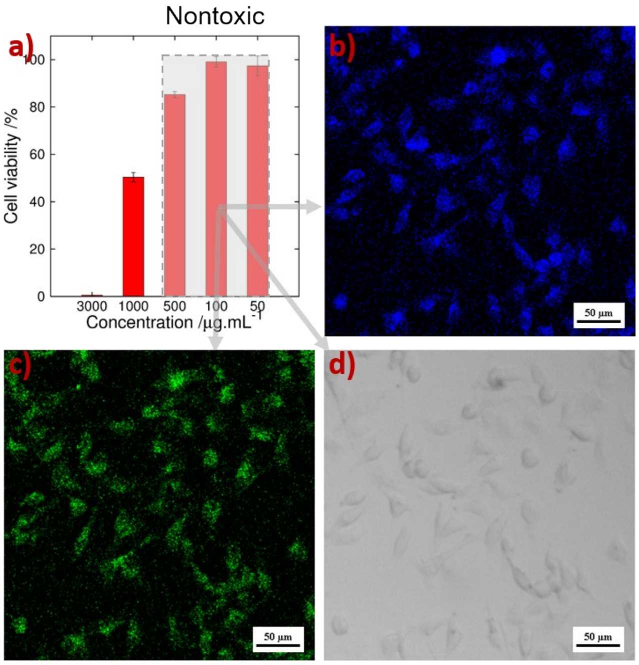

Figure S12. a) Cell viability of Hela cells incubated with different concentration carbon dots via CCK-8 assay and confocal fluorescence microphotographs of Hela cells incubated with ACD@HATU: b) λ_{ex} = 408 nm, c) λ_{ex} = 488 nm and d) bright field.
